# Nano-Scale Modifications of Amniotic Membrane Induced by UV and Antibiotic Treatment: Histological, AFM and FTIR Spectroscopy Evidence

**DOI:** 10.3390/ma14040863

**Published:** 2021-02-11

**Authors:** Simona Cavalu, George Roiu, Ovidiu Pop, Denisa A. Petricas Heredea, Traian Octavian Costea, Claudia Florida Costea

**Affiliations:** 1 Faculty of Medicine and Pharmacy, University of Oradea, 10 P-ta 1 Decembrie, 410073 Oradea, Romania; scavalu@uoradea.ro; 2 Advanced Materials Research Laboratory, University of Oradea, 1 University Street, 410087 Oradea, Romania; tcostea@uoradea.ro; 3 Department of Ophthalmology, “G.T. Popa” University of Medicine and Pharmacy Iasi, 16 University Street, 700115 Iasi, Romania; claudia.costea@umfiasi.ro

**Keywords:** amniotic membrane, FTIR spectroscopy, immunohistochemistry, AFM, UV light, antibiotic, pterygium surgery

## Abstract

The efficiency of amniotic membrane (AM) transplantation in different types of ocular surface disorders is due to its outstanding properties such as antifibrotic, antibacterial, anti-inflammatory and antiangiogenic, working as a versatile scaffold to promote corneal tissue epithelialization. A proper preparation, preservation and clinical application are crucial for the best outcomes in the treatment of different severe ocular disorders, taking into account its fragility. In this context, by combining high-sensitivity tools such as atomic force microscopy (AFM) and Fourier transform infrared (FTIR) spectroscopy with histological and immunohistochemical examination, we aimed to investigate the ultrastructural modifications of the amniotic membrane (AM) upon UV exposure and/or antibiotic treatment, with relevance for clinical applications in ocular surface surgery. From the morphological point of view, we noticed a loss of cuboidal cells in the basal membrane, accompanied by the splitting of collagen fibers upon UV and/or gentamicin treatment, while structural alteration of proteins was evidenced by the FTIR quantitative analysis of the secondary structure. A decrease in α-helix and β-sheet content, accompanied by increased content in less ordered structures (turns, random and side chains), was noticed after all the treatments. At the nano-scale, AFM details showed modifications of collagen fibrils in terms of their thickness and network compaction upon gentamicin and/or UV treatment. The enzymatic digestion assay demonstrated that UV exposure significantly reduces the degradation rate of the AM, while gentamicin treatment promotes an accelerated enzymatic digestion upon UV exposure. In order to highlight the clinical impact of the research, a clinical case is presented showing the relevance of amniotic membrane transplantation in pterygium surgery.

## 1. Introduction

**Figure 1:**
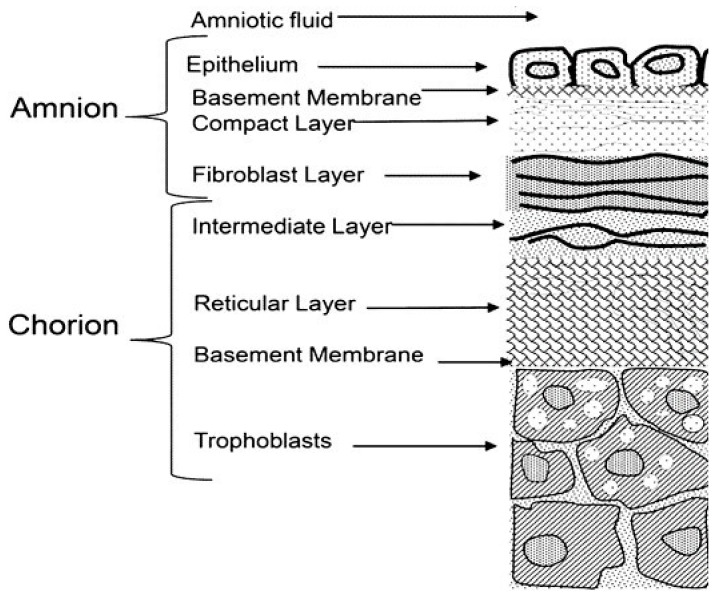
Schematic representation of the layered structure of the amniotic membrane (AM).

The structural and ultrastructural characteristics of the amniotic membrane (AM) along with its biological properties recommend this natural biomaterial as a support matrix for tissue regeneration, including corneal and conjunctiva surface reconstruction. It is not a substitute, but rather a substrate upon which the epithelial cells can easily grow, differentiate and migrate, helping tissue to regenerate [1]. The placental amniochorionic membrane comprises the inner layer, which is in direct contact with amniotic fluid (amniotic membrane), and the outer layer (chorion), which separates the amnion from the uterus, maternal blood and maternal side of the decidua (Figure 1).

The amniotic membrane itself is a thin, transparent, tough, avascular architecture, consisting of three layers: (i) an epithelial monolayer; (ii) a basement membrane; and (iii) an avascular stroma. Histological details reveal that the epithelium consists of metabolically active cuboidal cells, uniformly arranged on the basement membrane [2,3], and is comprised mostly of collagen (type III, IV, V), fibronectin and laminin. The stroma itself is also a layered structure: (i) a compact layer—which is the main fibrous skeleton of the AM, consisting of collagen (types I, III, V, VI) and fibronectin; (ii) a fibroblast layer, composed of fibroblast cells, collagen (types I, III, IV), fibronectin, laminin and nidogen; and (iii) a spongy layer containing mainly collagen (types I, III, IV) and proteoglycans [2]. Due to its layered structure, the AM can be easily separated from the chorion by means of blunt dissection. Not only the layered structure but also the biological properties of the AM provide suitable features for potential tissue engineering applications: anti-inflammatory, antimicrobial, antiviral and antifibrotic properties and the low immunogenicity of HAM (Human Amniotic Membrane) demonstrate a favorable environment for cellular attachment and expansion through an in vivo or in vitro approach [4,5,6,7,8]. Both the epithelial and mesenchymal amniotic cells are pluripotent stem cell reservoirs, while the matrix of the AM is very rich in growth factors such as keratinocyte growth factors (KGFs), fibroblast growth factors (FGFs), transforming growth factor beta (TGFβ), nidogen growth factors (NGFs) and epidermal-derived growth factor (EDGF) [7].

In recent decades, a special attention was paid to applications of the AM in ophthalmic surgery, taking into account the similarities between the AM and the conjunctiva and cornea in terms of its collagen composition and other proteins such as fibronectin and laminin. Various ophthalmic disorders, such as corneal ulcer, corneal perforation, chemical burns, pterygium, infectious or vernal keratitis and bullous keratopathy, have been successfully treated and the clinical results are well documented [6,7,8,9,10]. Currently available commercial AM products are either cryopreserved (−80 °C) as fresh membranes or dried, γ-sterilized, denuded membranes [11]. However, the native intact AM has been found to contain higher levels of growth factors compared to the denuded AM [11,12]. The denuded AM is often used as a culture substrate for limbal epithelial cells using both allogeneic and autologous explants [13]. For this purpose, crosslinking of the AM is necessary, and hence different crosslinking strategies have been used in order to increase the thermal and mechanical stability of the AM for the culture of epithelial cells, including glutaraldehyde, carbodiimide and UV radiation crosslinking [14]. In particular, with respect to the UV crosslinking procedure, it was demonstrated that the biostability of collagenous tissue strongly depends on the number of crosslinked structures, which are closely related to the UV exposure time [10]. Optimal crosslinking of collagen is essential for collagen binding to its receptors. The matrix permeability is drastically affected by the number of crosslinks per unit mass of the photo-crosslinked AM [15].

Although, with available modern preservation techniques, the membranes are now able to be stored for several months, allowing for scheduled surgical intervention, there is currently a debate over whether the cellular fresh, cryopreserved or dried, denuded form of the AM is considered a better substrate for promotion of epithelization in ocular surface reconstruction. While cryopreserved AMs retain the native architecture of the extracellular matrix and maintain the key biological signals, the dehydrated ones seem to be susceptible to damage in the basal lamina as a result of cell removal [11,12,13,14,15,16]. Therefore, AMs’ proper preparation, preservation and clinical application are crucial for the best outcomes in the treatment of different severe ocular disorders.

Individual post-surgery regimes using topical antibiotics in the form of eye drops are usually prescribed for adjunctive therapy, in order to prevent bacterial keratitis. Commercially available topical antibiotics for this purpose include fluoroquinolones, gentamicin, ciprofloxacin and clarithromycin [17], but some of these may also affect the structural properties of the AM in terms of the hydration recovery of proteins, which has often been interpreted as the main cause of collagen maturation and aging [18]. Post-surgery exposure to UV may also influence the success of ocular surface surgery, knowing the UV influence on the structural and mechanical properties of collagen fibrils [16]. Many studies have been devoted to successful outcomes of either fresh or dehydrated membranes, performed in vivo or in vitro, but there is a lack of information related to the post-surgical conditions and potential detrimental effects of antibiotics as well as UV exposure. Hence, one of the goals of our study was to investigate to what extent the antibiotic concentration will influence the structural properties of amniotic membranes prepared for corneal reconstruction.

In the context of the above-mentioned debate, the overall aim of our work was to investigate the ultrastructural modifications of the AM upon UV exposure and/or antibiotic treatment by combining the histological/immunohistochemical examination with high-sensitivity atomic force microscopy (AFM) measurements and Fourier transform infrared (FTIR) spectroscopy. Moreover, an in vitro enzymatic assay (collagenase digestion) was performed, along with a clinical case presentation (pterygium) requiring corneal surgery and resurfacing with HA showing the most important outcome and advantages of this technique.

## 2. Materials and Methods

### 2.1. Procurement and Preparation of Biological Tissue

The research protocol was performed in agreement with the ethical standards of the Helsinki Declaration and approved by the Ethical Committee of the University of Oradea, Romania (ref. nr. 06/15.10.2020). The biological tissue was obtained under strict aseptic conditions, from a patient who had undergone cesarian section at full term, with informed consent. Under a laminar flow hood, the membrane was washed with sterile physiological saline to remove blood clots, separated from the chorion by blunt dissection and peeled. Then, the AM was cut into small-size (5 cm × 5 cm) specimens by using a sterile scalpel; each piece was again washed three times with sterile distilled water and flattened on individual Petri dishes, to which 5 mL PBS (Phosphate Buffer Saline) was added and stored at −20 °C until further treatments and preparation for histological examination, FTIR and AFM measurements.

### 2.2. Antibiotic and UV Treatment

The AM specimens were divided into 4 groups and the following treatments were applied:

i. Antibiotic treatment: Specimens were allowed to interact with the gentamicin injectable solution (KRKA, Novo Mesto, Slovenia) concentrations of 40 and 80 mg/mL, for 1 h, and then they were washed with PBS, rinsed with sterile distilled water, flattened on a cellulose support and stored in a refrigerator until FTIR and AFM investigations. The samples were labeled AG40 and AG80, respectively, according to each concentration.
ii. UV treatment: Specimens were exposed to UV in air using a GL4 germicidal lamp (Philips TUV 6W G6) at no more than 254 nm, for 1 h, and then kept in a refrigerator until further investigations. The samples were labeled AUV.
iii. Combined antibiotic/UV treatment: Immediately after gentamicin treatment (concentration 40 mg/mL), specimens were exposed to the UV treatment described above and then kept in a refrigerator until further investigations. The samples were labeled AGUV.
iv. The control sample was the natural amniotic membrane without any treatment, labeled AMN.

The above treatments were carried out in triplicate.

### 2.3. Histological and Immunohistochemical Examination

For the histological and immunohistochemical examination, the specimens were fixed in 4% formaldehyde immediately after the antibiotic or/and UV treatment. The immunohistochemical analysis was performed on 4 μm-thick sections prepared from a formalin-fixed paraffin-embedded block by using an Autostainer Link 48 (Agilent Technologies, Santa Clara, CA, USA). Immunohistochemical assays were used on paraffinized slides to perform target retrieval. The slides were developed using a DAB (3,3′-diaminobenzidine) detection kit and counterstained with hematoxylin. The sections were incubated with anti-Collagen IV (clone CIV 22), mouse monoclonal antibody (Agilent Technologies, Santa Clara, CA, USA). For each case, positive control slides were used, prepared from appendix tissue against a glandular basement membrane. Negative control was performed by omitting the primary antibody.

### 2.4. FTIR Spectroscopy

For the FTIR measurements, the specimens were left to dry overnight in a refrigerator, at 4 °C. A Shimadzu FT 8400 S (Shimadzu Co., Kyoto, Japan) FTIR spectrophotometer was used, equipped with an MIRacle ATR accessory (ZnSe crystal), operating in the range 400–4000 cm^−1^, with the following spectral acquisition parameters: wavelength resolution 2.00 cm^−1^, Happe–Genzel apodization function, 10 scans/spectrum. Data processing was performed using Origin 8 software with a Gaussian–Lorentzian function applied for fitting parameters. The amide I spectral band was baseline-corrected, and the area was normalized and deconvoluted in the interval 1600–1700 cm^−1^. The percentage of protein secondary structures (α-helix, β-sheets, turns, unordered and side chains) was calculated based on the area under each peak, and the assignments of the components were conducted according to the literature [19,20,21]. Results were represented as mean ± standard deviation (SD) for three independent experiments. A probability level of *p* < 0.05 was considered statistically significant, and the analysis was conducted by Student’s *t*-test.

### 2.5. AFM Measurement

Atomic force microscopy (Agilent 5500 AFM, Agilent Technologies, Santa Clara, CA, USA) was applied to obtain the ultrastructural details of the amniotic membrane, air-dried, after different treatments. The thickness of individual collagen fibrils was measured by recording their profile. The specimens were fixed on glass plates with a double-sided tape. The scanning was performed at room temperature and a normal humidity level (50%), in acoustic mode (taping mode), in which the AFM tip was oscillating slightly below its resonance frequency of 317.14 kHz and scanned the selected area with a speed of 5.361 m^−6^/s at a resolution of 512 × 512 data points, in contact mode, in which the AFM tip maintained a close contact with the surface of the sample while scanning the selected area with a speed of 2.160 m^−6^/s at a resolution of 512 × 512 data points.

### 2.6. Enzymatic (Collagenase) Degradation Assay

The AM specimens prepared according to Section 2.1 and Section 2.2 were allowed to dry, and then they were weighted (balance model FA-G Want Balance Instr. Changzhou, China), immersed individually in a 0.1% collagenase solution (Sigma–Aldrich. St. Louis, MO, USA) with pH = 7 and incubated at 37 °C (incubator Model MCO-5 AC, Sanyo/Panasonic Biomedical, York, UK) in static conditions, under a flux of 5% CO_2_. After different time intervals (12, 24, 36, 48, 60 and 72 h), the specimens were carefully removed and allowed to dry completely and weighted again. The weight of the remaining mass was expressed as a percentage (mean value ± SD) and the statistical significance was measured by an ANOVA test. A *p*-value of less than 0.05 was considered significant.

### 2.7. Clinical Case

A case of recurrent pterygium is presented, in which the AM (fresh, preserved at −20 °C) was successfully applied in order to reconstruct the corneal surface, after the surgical removal of the damaged tissue. The patient (30 years old, male, living in a rural area) was previously informed about the surgical procedure and he gave his informed consent for inclusion in the study, in accordance with the Declaration of Helsinki and the research protocol approved by the Ethical Committee of the University of Oradea, Romania (ref. nr. 06/15.10.2020). Before surgery, a complete ophthalmologic examination including measurement of intraocular pressure, visual acuity and biomicroscopy was performed.

### 2.8. Statistics

Results were expressed as mean ± standard deviation for three independent experiments, using Student’s *t*-test in the case of FTIR measurements, while one way analysis of variance (ANOVA) was employed for the enzymatic digestion assay. In both cases, significance was accepted with *p* < 0.05. 

## 3. Results

### 3.1. Histological Examination

**Figure 2:**
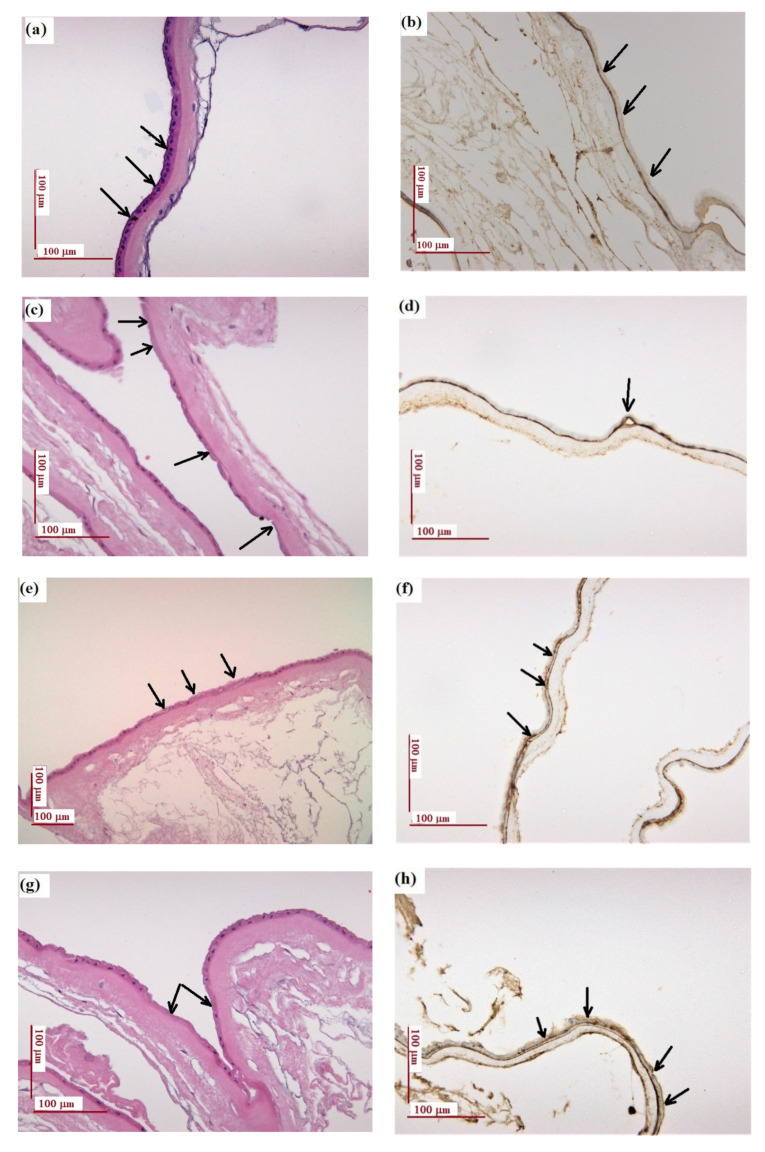

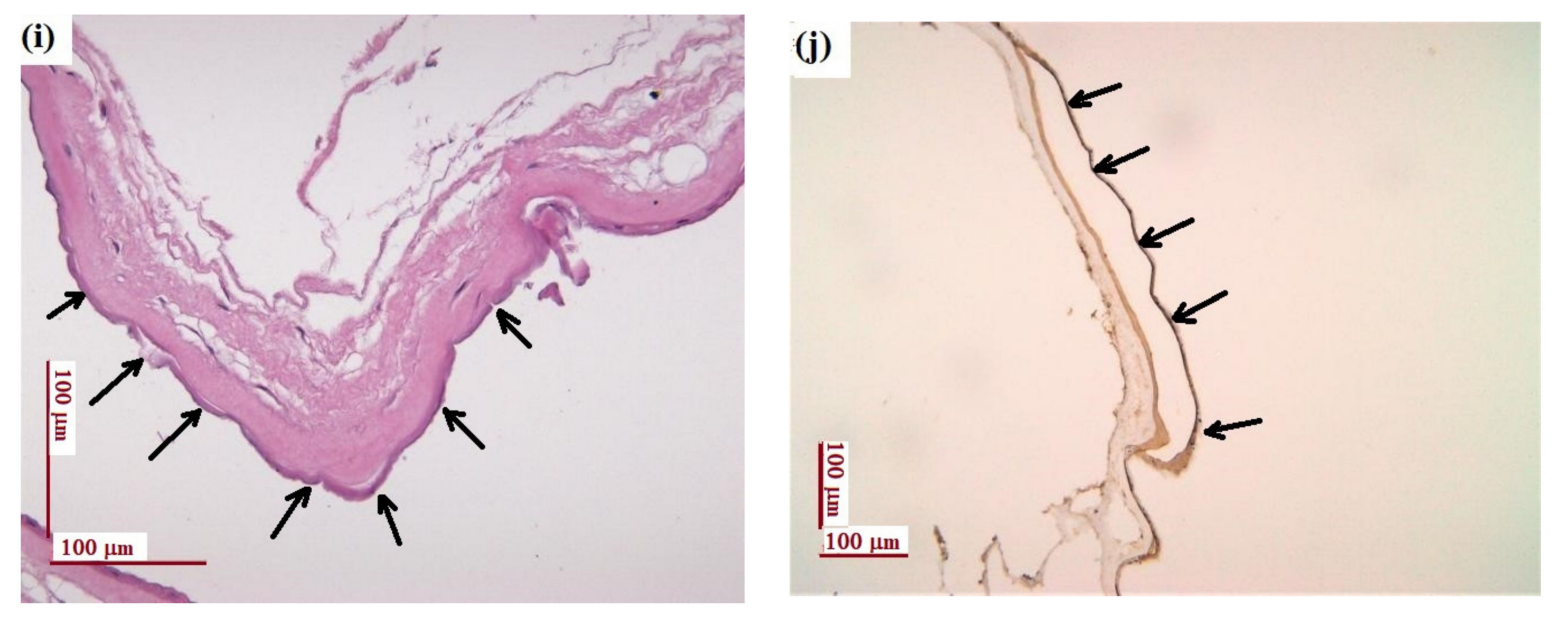
Histological and immunohistochemical examination: (**a**,**b**) natural amniotic membrane (AMN); (**c**,**d**) amniotic membrane exposed to UV for 1 h (AUV); (**e**,**f**) amniotic membrane treated with gentamicin (40 mg/mL) (AG40); (**g**,**h**) amniotic membrane treated with gentamicin 80 mg/mL (AG0); (**i**,**j**) amniotic membrane treated with gentamicin 40 mg/mL and exposed to UV for 1 h (AGUV). Left panel: H&E staining; right panel: immunohistochemistry staining of collagen IV (antibody clone CIV 22). Scale: 100 μm.

The details of H&E staining reveal a uniform layer of cubic cells displayed on a basement membrane of the AMN sample (Figure 2a), while the expression of collagen IV in the basement membrane is continuous and dense (Figure 2b). After exposure to UV light, a moderate loss of cubic cells can be seen in several spots (Figure 2c), with a dense and continuous expression of collagen IV; a splitting of about 8 μm in collagen fibers was detected in a single spot (Figure 2d). The cuboidal cells slightly changed their size and shape after the treatment. The loss of cubic cells can be noticed also after gentamicin treatment (Figure 2e), accompanied by a splitting of about 3 μm in collagen fibers, over a relatively long distance (Figure 2f). By applying a double gentamicin concentration, the basement membrane maintained its integrity, but the loss of cubic cells can be noticed in a higher number of foci (Figure 2g), while the splitting of the collagen fibers is more significant, the distance between the two expressions being variable, from 8 to 20 µm (Figure 2h). The UV exposure of gentamicin-treated specimens showed extensive loss of cubic cells (Figure 2i), concomitantly with a significant splitting of collagen fibers (about 70 µm) over a long length, as shown by the expression of collagen IV (Figure 2j).

### 3.2. FTIR Spectroscopy

**Figure 3:**
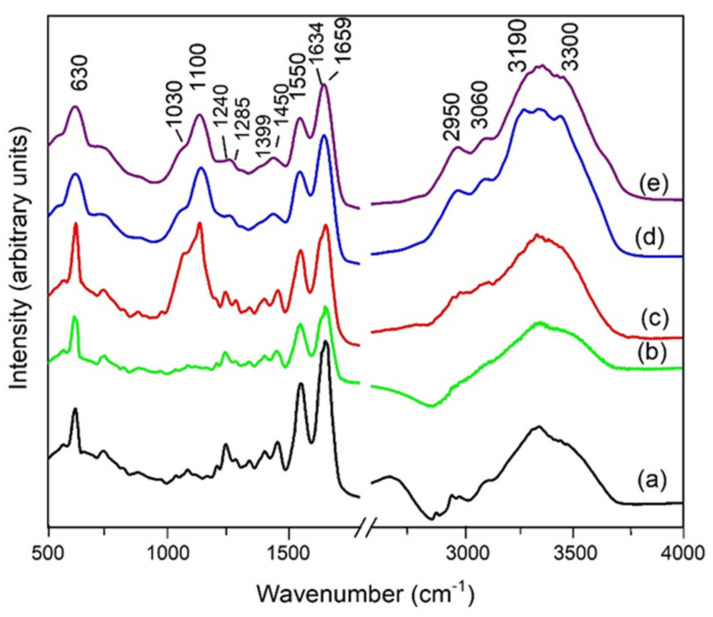
FTIR spectra of AM specimens after treatment with antibiotic and/or UV: (**a**) natural AM without any treatment (AMN); (**b**) AM exposed to UV for 1 h (AUV); (**c**) AM treated with gentamicin (40 mg/mL) and exposed to UV for 1 h (AGUV); (**d**) AM treated with gentamicin 40 mg/mL (AG40); (**e**) AM treated with gentamicin 80 mg/mL (AG80).

**Figure 4:**
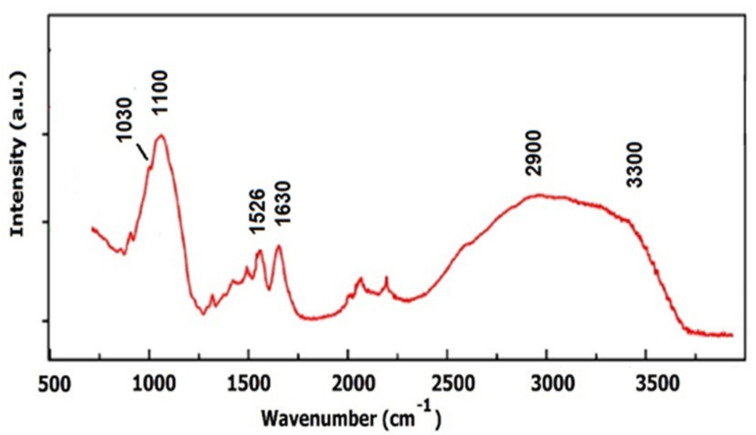
FTIR spectrum of gentamicin.

The FTIR spectra recorded in the range 400–4000 cm^−1^ are presented in Figure 3 for AM specimens belonging to each treatment group, while in Figure 4, the FTIR spectrum of pure gentamicin is presented.

In the low-wavenumber region, the absorption band around 1659 cm^−1^ corresponds to the amide I protein absorption band, being assigned to the C=O stretching mode, while the absorption band around 1550 cm^−1^ corresponds to amide II absorption attributed to the N–H bending mode and C–N stretching mode. Amide III protein absorption is located between 1240 and 1300 cm^−1^, as a result of the in-phase combination of C–N stretching and N–H in-plane bending, and also the contribution from C–C stretching and C=O bending vibrations. The band close to 1450 cm^−1^ is probably associated with C–H bending modes and the amide A band (NH stretching) observed in the high-wavenumber region, between 3190 and 3300 cm^−1^, suggesting a low water content in the sample [21,22,23]. The peaks at 1399 and 630 cm^−1^ are attributable to the carboxylate ion and the C=O planar deformation. The band at 1100 cm^−1^ is attributed to the phosphodiester group of nucleic acids and glyco- and phospho-lipids, while the shoulder at 1030 cm^−1^ represents the fingerprint of gentamicin [17], as emphasized in Figure 4. In the higher-wavenumber region, the interval 3190–3300 cm^−1^ is assigned to O–H and C–H bending vibrations, while the band at 2950 cm^−1^ can be assigned to the asymmetric stretching mode of the CH_3_ group. The O–H stretching region can be correlated to the hydrogen bond network around the protein, assuming that a certain hydration level still exists after the drying procedure [19,24]. However, water deprivation and restitution might have some consequences on the macromolecular structure. As a general behavior, after antibiotic and or/UV treatment, the main fingerprints of proteins shifted towards higher wavenumbers (with about 15 cm^−1^), concomitantly with changes in the relative intensity of the amide I, II and III bands.

Computational processing of the FTIR spectra is required because FTIR spectroscopic features contain thousands of singular spectra. For this reason, a fitting procedure was applied in order to evaluate, from the quantitative point of view, the structural modifications of the protein matrix, after UV and antibiotic treatment. The most important structural changes can be evidenced through the computational analysis of the amide I band, which is commonly used to evaluate different secondary structure elements, taking into account that the amide I band is composed of superimposed absorption bands from the α-helix (1659 cm^−1^), β-sheets (1628 cm^−1^), turns (1678 cm^−1^), unordered or random coil contributions (1646 cm^−1^) and side chains (1613 cm^−1^) [21,23,24,25,26].

**Figure 5:**
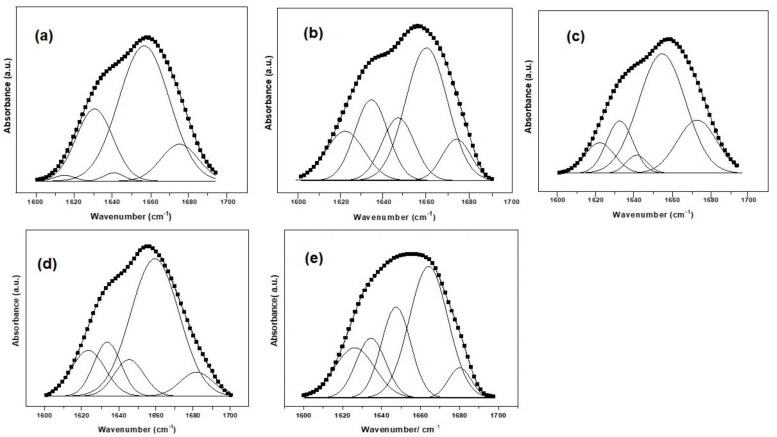
Computational fitting of the amide I FTIR spectroscopy absorption band after different treatments: (**a**) natural membrane (AMN), (**b**) 1 h exposure to UV (AUV), (**c**) gentamicin treatment (40 mg/mL) with 1 h exposure to UV (AGUV), (**d**) gentamicin treatment 40 mg/mL (AG40) and (**e**) gentamicin treatment 80 mg/mL (AG80).

**Figure 6:**
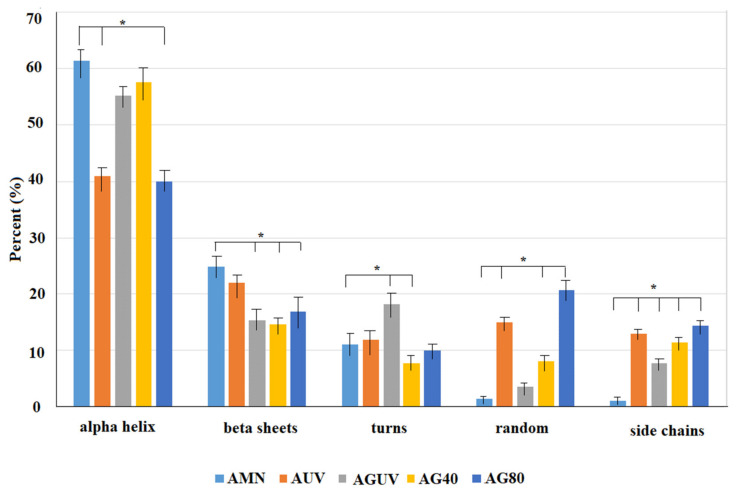
Quantitative analysis (percent) of the collagen secondary structure in the amniotic membrane after different treatments: natural membrane (AMN), 1 h exposure to UV (AUV), gentamicin treatment (40 mg/mL) and 1 h exposure to UV (AGUV), gentamicin treatment 40 mg/mL. * *p* < 0.05 was considered significant.

The fitting procedure assumed a Gaussian shape for the amide I band envelope and individual contributions. Each component of the secondary structure corresponds to different C=O stretching frequencies, resulting in different band positions. The sum of the areas under each peak represents the total amount of secondary structures in the protein. The computational fitting of the amide I vibrational band of the natural amniotic membrane and after antibiotic treatment is presented in Figure 5a–e, while the quantitative assessment of each secondary structure is presented as a diagram in Figure 6.

### 3.3. Nanotopography: AFM Examination

**Figure 7:**
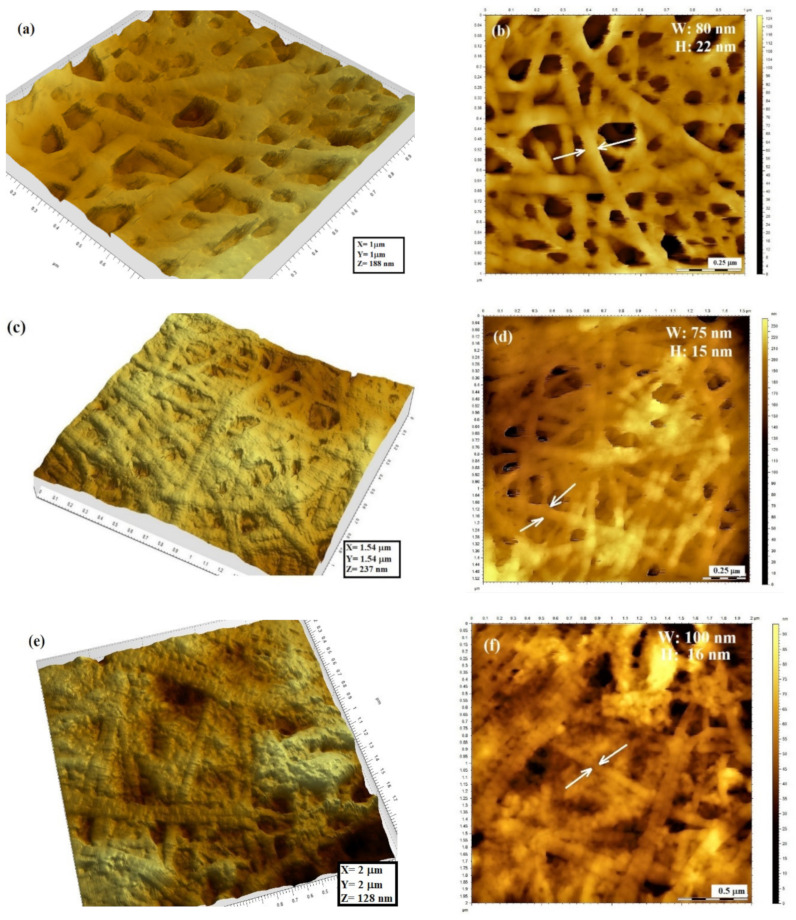

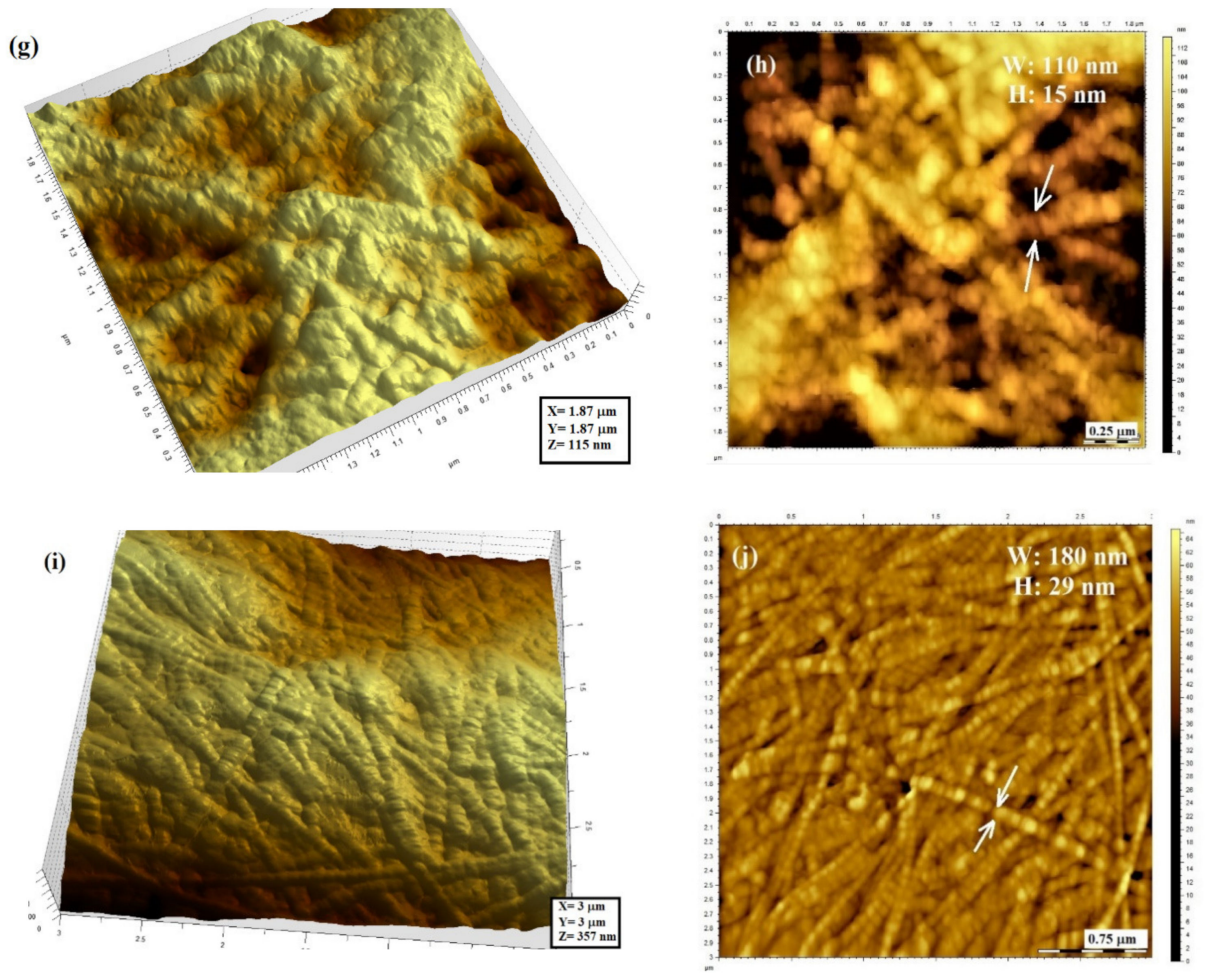
AFM examination of the amniotic membrane after different treatments (3D and 2D images) indicating the features of a single collagen fibril: (**a**,**b**) natural membrane (no treatment) (AMN); (**c**,**d**) membrane exposed to UV (AUV); (**e**,**f**) membrane exposed to gentamicin treatment 40 mg/mL (AG40); (**g**,**h**) membrane exposed to gentamicin treatment 80 mg/mL (AG80); (**i**,**j**) membrane exposed to gentamicin and UV treatment (AGUV). The profiles of a single collagen fibril exposed on the surface of the amniotic membrane after different treatments are presented in the Supplementary Materials (Figure S1).

The 3D and 2D topographic features of the amniotic membrane after different treatments are presented in Figure 7, also indicating the details of a single collagen fibril exposed on the surface (W = width; H = height).

As presented in Figure 7, the structural collagen fibrils comprising the AM membrane are fully exposed at the surface and randomly arranged in a 3D network. According to Figure 7a,c, the thickness of a single collagen fibril in the natural AM, untreated (AMN), is about 80 nm in width and 22 nm in height. Upon different treatments, the surface roughness was modified, and a more compact structure was noticed, accompanied by changes in the fiber thickness. The most significant change can be noticed for the AGUV sample, reaching 180 nm in width and 29 nm in height. The sole UV treatment does not show significant modifications in terms of fibril thickness. When gentamicin was applied, an increase in the fibril width was noticed, with an insignificant influence of the gentamicin concentration. Although the details of the D-bands of a single collagen fibril are not very clear in our AFM images, we can notice that the D-periodicity of collagen fibrils is not significantly altered by UV radiation or the gentamicin concentration. Further, the length of collagen chains seems to be well preserved within the 3D network.

### 3.4. Collagenase Digestion Assay

**Figure 8:**
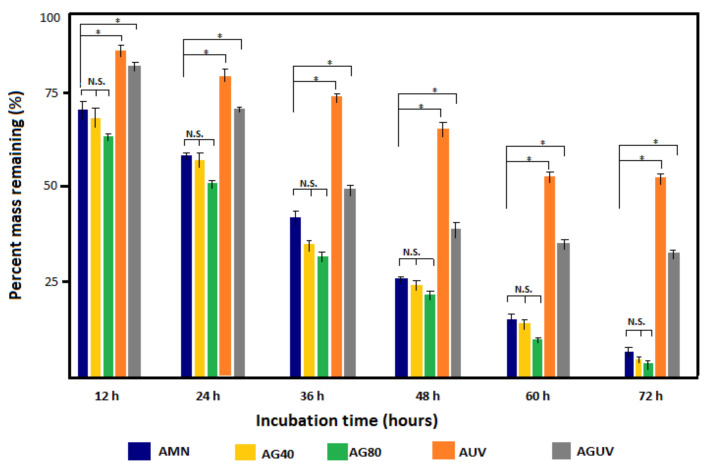
Collagenase digestion of the amniotic membrane after different treatments: natural membrane (AMN), gentamicin treatment with 40 mg/mL (AG40), gentamicin treatment with 80 mg/mL (AG80), 1 h exposure to UV (AUV) and 1 h exposure to UV after gentamicin treatment (AGUV). * *p* < 0.05 was considered significant; N.S.—non-significant.

Figure 8 presents the results of the collagenase digestion of the amniotic membrane prior to any treatment and following the UV and/or gentamicin treatment monitored for 72 h. Within the first 12 h, a drastic degradation of AMN, AG40 and AG80 specimens was noticed, compared to AUV and AGUV specimens, and this behavior had a similar, continuous trend for the entire time interval. At each time point, insignificant modifications were noticed between gentamicin-treated samples and untreated ones, no matter the antibiotic concentration. After 72 h of digestion, less then 8% of AMN, AG40 and AG80 remained undigested, while the UV-treated specimens (AUV and AGUV) showed enzymatic resistance of 55% and 32%, respectively, in terms of the mass remaining. By comparing both UV-treated specimens, it can be noticed that gentamicin strongly influences the degradation behavior, which is reflected by the mass remaining when comparing AUV and AGUV percentages at each time point. The difference is more obvious in the time interval 36–72 h. However, for the last 12 h, a slower degradation of AUV and AGUV specimens was noticed.

### 3.5. Clinical Case

**Figure 9:**
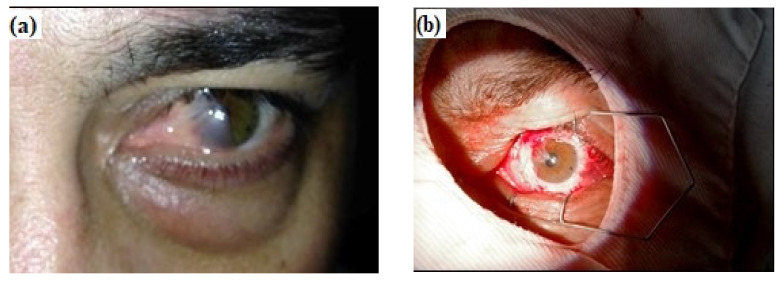

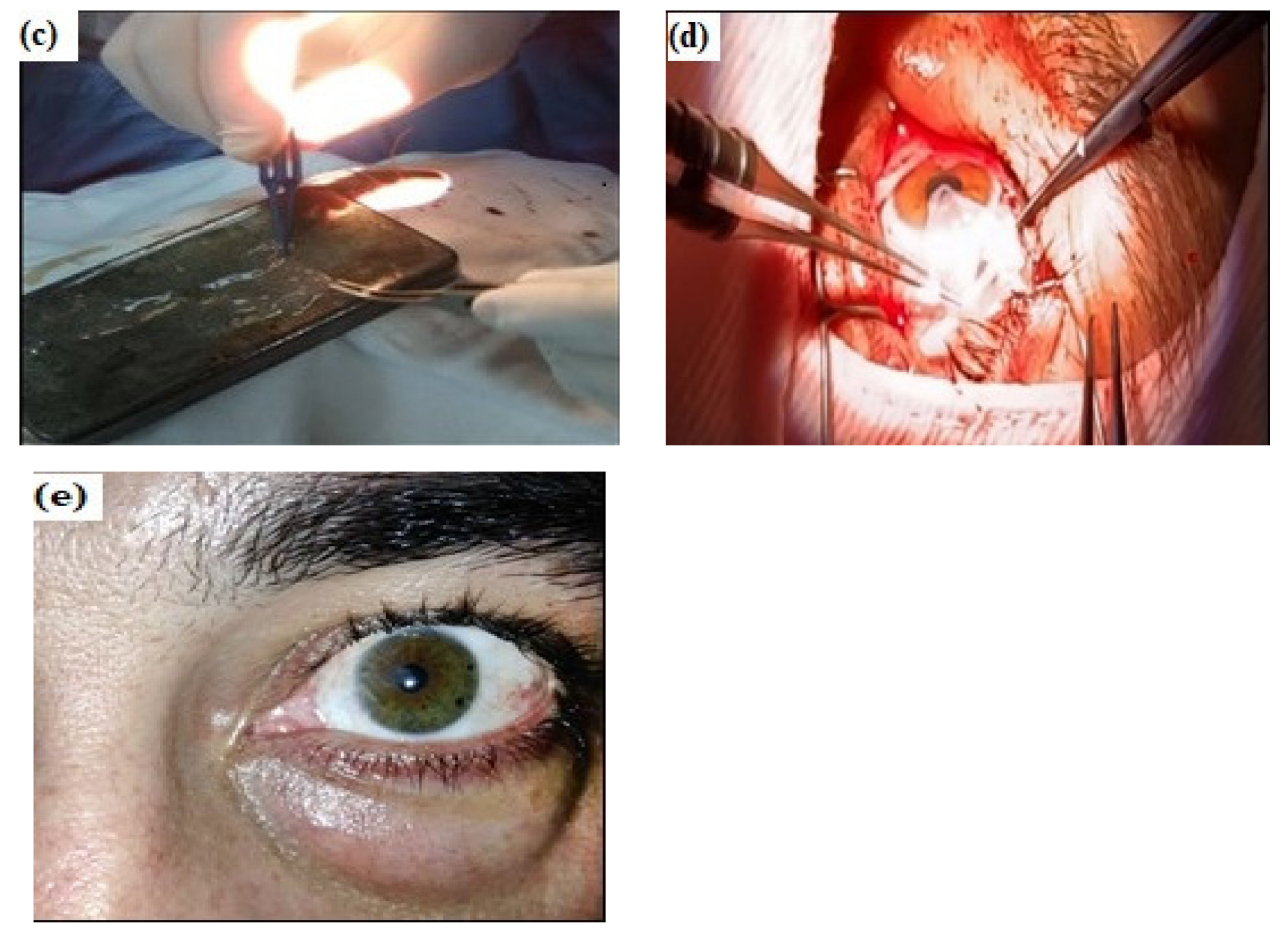
Amniotic membrane used in ophthalmologic surgery to cover the remaining tissue defect due to the grade 3 recurrent pterygium excision: (**a**) grade 3 recurrent pterygium, preoperative appearance; (**b**) intraoperative aspects (the same patient); (**c**) intraoperative preparation of the amniotic membrane fragment to be applied on the remaining defect following recurrent pterygium excision; (**d**) intraoperative appearance—application of the AM fragment on the remaining defect and 10.0 thread suturing to the bulbar conjunctiva of the fragment; (**e**) the aspect of the patient’s cornea four weeks after surgery (from private collection of Claudia Florida Costea).

Figure 9a–e presents a clinical situation in which the amniotic membrane was used in ophthalmologic surgery, as a grafting biomaterial, to cover the remaining tissue defect due to the pterygium excision (or its recurrence).

The standard protocol during pterygium surgery was applied, involving the peeling of the abnormal corneal tissue, followed by blunt dissection of the pterygium head, with caution to not affect the corneal stromal tissue. The excision of adjacent residual pterygium tissue was also performed, along with the fibrovascular tissue. The AM (preserved at −20 °C) was cut to fit over the corneal defect, with the stromal side down, in order to promote epithelial adhesion and rapid healing. Immediately after surgery, the eye was patched and shielded. The post-surgical treatment consisted of gentamicin sulfate 3%, dexamethasone and artificial tear instillations and a recommendation of total protection against UV radiation exposure. The patient was examined each day in the first week, and then once a week. After four weeks, complete healing of the corneal tissue was noticed.

## 4. Discussion

The clinical success of the AM in the reconstruction of ocular surfaces is related to its composition, as the AM contains and releases a remarkable mixture of growth factors and cytokines that facilitate the proliferation and differentiation of epithelial cells, concomitantly with reducing the inflammatory response by inhibiting protease activity [27,28,29]. It has been demonstrated that the AM could promote ocular surface tissue healing of persistent epithelial defects (corneal ulcers, pterygium, bullous keratopathy, symblepharon and eye burns), provides a substrate for cell growth, has antimicrobial effects and functions as a biological bandage [3,29,30]. As described in the literature, there are three different surgical techniques: graft or inlay, patch or overlay and combined multilayer techniques [31]. In the graft or inlay technique, the AM is administered as a permanent basement membrane substitute, being integrated in the host tissue and acting as a scaffold for epithelial cells to grow. This technique is usually applied to persistent epithelial defects, corneal ulceration or conjunctival tumors, by suture. In the patch or overlay technique, the AM is placed temporarily on the ocular surface, acting as biological bandage, but not being integrated into the host. In this case, the epithelium is expected to grow underneath rather than over the top, and the AM will disassociate from the ocular surface after a certain time [32]. Regardless the surgical technique employed, topical antibiotic application is required in order to eliminate the risk of infectious keratitis. Eye drops are preferred in most cases of multiple bacterial keratitis, consisting of cefalozin, vancomycin, tobramycin, gentamicin or fluoroquinolones, which have been approved by the Food and Drug Administration for the treatment of bacterial keratitis [33,34]. Fortified eye drops, as a recent trend in antimicrobial therapy of bacterial keratitis, relate to formulations with a very high concentration, in order to fight against increasingly resistant Gram-negative bacteria (*S. aureus, P. aeruginosa*), which are known for their ability to destroy the corneal stroma [34,35]. In vitro and in vivo experiments suggest that the antibiotic enters the cornea via direct diffusion rather than through the precorneal tear film [36]. In some animal model experiments, crystalline corneal epithelial deposits were noticed with fortified topical eye drop instillations, and a possible delay of re-epithelialization was assumed [37].

Based on these previous results, the overall goal of our study was to investigate the structural and morphological modifications of the collagen matrix in the AM, by the means of histological observation, FTIR spectroscopy and AFM images, upon treatment with gentamicin and exposure to UV-C radiation. In particular, we also aimed to investigate to what extent the antibiotic concentration will influence the structural properties of the amniotic membrane prepared for corneal reconstruction.

The native membrane (AMN) presents a typical epithelial cell layer, as a single, continuous layer of cuboidal and columnar cells on a densely eosinophilic basement membrane, as revealed in H&E staining (Figure 2a), which is in agreement with previously reported data [2,3,9]. Moreover, the immunohistochemical staining revealed that the basement membrane is very rich in collagen IV, showing a dense and continuous expression. The loss of cubic cells was noticed after gentamicin treatment, regardless of the antibiotic concentration, accompanied by the splitting of the collagen fibers while maintaining the integrity of the basement membrane. When UV treatment was applied to the natural membrane, morphological changes were noticed in terms of cell size and a tendency of collagen fibers to split in double chains. Drastic changes in the morphology of the AM were noticed when antibiotic treatment was followed by UV exposure for 1 h (specimen AGUV). The loss of cuboidal cells was observed, to a large extent, on the length of the basal membrane, while the splitting of collagen fibers was noticed over a very long length, the distance between the two expressions being larger compared to UV treatment alone, as evidenced by the immunohistochemical staining.

In order to obtain deeper details about the possible denaturation of collagen, we further performed FTIR investigations and deconvolution of the amide I absorption band and assessed structural changes in the molecules. Collagen is the principal structural component of the AM, having a unique structure characterized by three polyproline II-like helical chains, packed in a left-handed triple helix which is stabilized by glycine at every third residue. The triple helices assemble to form fibrils, which are then aligned laterally to form bundles and fibers. Under electron microscopy, the collagen molecules within the fibrils present a D-band pattern with periodic gaps and grooves, of about 67 nm, which are correlated to pathological conditions and the fibril mechanical strength [38,39]. As demonstrated by previous research [15,37,38], the native collagen triple helix is most sensitive to UV-254 nm radiation, reflected in changes in the primary and secondary structures and changes in the conformation, microstructure and material properties.

All the treatments applied in our study indicate a decrease in α-helix and β-sheet content, accompanied by increased content in less ordered structures, such as turns and random and side chains, while the position of the main FTIR fingerprints was slightly shifted toward higher wavelengths. The results are consistent with previously reported data [40], which evidenced the behavior of amide I band components in both native and denatured forms of collagen due to UV treatment, suggesting a helix–coil transformation of collagen. The triple-helical regions of collagen are known to be stabilized by hydrogen bonding and van der Waals attractions between imine residues on different chains [15]. Both chemical and UV crosslinking of collagen are associated with its denaturation due to the collapse of hydrogen bonds in polypeptides. Glutaraldehyde, chitosan, poly(2-hydroxyethyl methacrylate), riboflavin and carbodiimide were reported in the literature as efficient crosslinking agents for the AM [25,41,42], showing different degrees of denaturation.

On the other hand, the crosslinking process enhances the biostability of the AM matrices to support limbal epithelial cell growth and can significantly improve the mechanical properties of the collagen matrix [41]. Therefore, although the crosslinking may cause denaturation of the collagen matrix, in vitro experiments of cultures on modified AM samples demonstrated that epithelial progenitor cells were preserved more effectively and the benefits of crosslinking prevail in terms of AM functionality [15]. Moreover, some previous reports demonstrated that ex vivo expansion of limbal epithelial cells occurs at a faster rate on the decellularized AM (with sodium dodecyl sulfate) compared with the fresh, natural one [43]. However, according to some authors [24,42,44], a lower sheet/turn ratio after the treatment indicates inferior biocompatibility when compared to the native AM. Based on the quantitative results presented in Figure 6, the sheet/turn ratio has an average value of 2.27 for the AMN specimens, 1.83 for AUV, 1.88 for AG40, 1.70 for AG80 and 0.83 for AGUV specimens. The lowest value (0.83) indicates the lowest biocompatibility, which was noticed for AGUV specimens, the results being also supported by the histological observations.

Nanotopographic measurements on the surface of the native AM and after different treatments revealed the details of collagen fibrils in a 3D network, which is influenced by the antibiotic treatment and UV exposure. Stylianou et al. demonstrated that in the nano-scale range, the collagen topography and biological properties are affected by UV radiation, which may induce alterations in cell behavior [39]. By applying the AFM technique, they investigated the influence of UV irradiation time (1, 3 and 7 h corresponding to energy doses of 1.1–13.2 J/cm^2^) on the collagen film nano-topography. The authors demonstrated that for short irradiation times (10–60 min), the film morphology did not present any significant alteration in terms of roughness, while the periodicity of the D-band pattern was preserved. After longer irradiation times (3–7 h), the roughness was gradually reduced, while after 11 h, the surface was dramatically altered, and the D-band pattern was destroyed, demonstrating an irreversible denaturation process of the triple helix. Based on these results, the authors concluded that UV irradiation can be used as a process for manipulating and controlling the surface roughness of collagen-based materials. In our work, we found modifications in terms of nano-morphology upon 1 h irradiation of the natural AM, namely, a slight reduction in the single fibril thickness from 80 in width and 22 in height to 75 in width and 15 nm in height, while a more compact structure was achieved. An increase in fibril thickness was noticed upon gentamicin treatment, depending on the concentration: 100 width/16 nm height for AG40, and 110 width/15 nm height for AG80, the length of collagen chains being well preserved within the 3D network. Notable changes were identified when UV exposure was applied immediately after gentamicin treatment (AGUV), in terms of collagen chain fragmentation, accompanied by increased thickness of collagen fibrils (180 width/29 nm height), although the D-band pattern for short collagen fragments was not altered, which is in concordance with previously reported data [39,45,46]. However, we could not find in the literature any reference related to the antibiotic influence on ultrastructural changes of the amniotic membrane, except our previous work [18], in which we demonstrated that gentamicin treatment is more favorable compared to ciprofloxacin, by assessing the denaturation process using FTIR spectroscopy. In a recent study performed by Zhang et al. [41], the evaluation of ultrastructural changes of amniotic membrane fragility was assessed after UVA/riboflavin crosslinking. The authors showed that after crosslinking treatment, the AM underwent biomechanical and ultrastructural changes: the brittleness was increased, the hardness was enhanced and the bamboo-like fiber morphology was changed. Another study was performed in order to examine ultrastructural images of the surface morphology of the air-dried and gamma-sterilized AM observed under AFM and to quantify possible changes in epithelial cell size, intercellular gap size and surface roughness [46], but with no relation to the collagen matrix. In this case, AFM results confirmed that both gamma radiation and air drying caused a reduction in cell sizes (about 32.3% reduction in the average longest diameter), but the changes did not affect the gross morphology of the membrane.

The collagenase digestion assay performed in our study demonstrated that UV exposure significantly reduces the degradation rate of AM, including the prior gentamicin treatment, while the gentamicin concentration has an insignificant influence on the dissolution time. It is well known that a fresh amnion usually dissolves within 1 week [42,47], but the crosslinking procedures (by glutaraldehyde, carbodiimide, riboflavin, UV radiation) significantly increase resistance to collagenase digestion, especially in the anterior half of the cornea [14,25,41,42,47]. Photochemical crosslinking is a rapid and efficient process with little toxicity, while chemical crosslinkers (e.g., riboflavin and glutaraldehyde) act as a photomediator, creating free radicals in the presence of UV radiation, promoting the formation of new chemical bonds bridging amino groups in collagen fibrils and subsequently increasing mechanical stiffness [48]. Our results suggest that gentamicin weakens the UV crosslinking effect of the AM, as evidenced by an accelerated degradation of AGUV compared to AUV specimens. These results might have a significant importance for in vivo application of the AM as a substrate for the growth and development of endothelial cells. According to Spoerl et al., the poor biological stability of the AM graft is a main cause of failure and early detachment after surgical transplantation in corneal tissue [42,47].

In order to highlight the clinical impact of our research, a clinical case was presented showing the relevance of pterygium surgery using the AM. Pterygium is a common benign growth of the conjunctiva from the nasal side of the sclera. According to the literature [49], the autograft surgery can be employed in these cases, but it is time-consuming and the recurrence rate is relatively high. As an alternative, the amniotic membrane is often used in order to prevent pterygium recurrence, the effectiveness of this technique being demonstrated especially for the removal of pterygium associated with severe conjunctival disease [50]. The success or failure of this procedure can be evaluated based on the purpose of surgery and by several criteria: resolution of inflammation, relief of symptoms, restoration of the corneal epithelium, recurrence. Recurrence was defined as the presence of fibrovascular proliferative tissue crossing the limbus. The selected clinical case presented in Section 3.5 was a grade 3 recurrent pterygium in which the natural AM (preserved at −20 °C) was successfully applied to cover the remaining defect following pterygium excision. In this case, by using the standard protocol, the membrane displayed scaffold-like properties, providing a healthy and intact basal membrane on which the patient’s cells were able to proliferate. Further, measures were taken to prevent AM damage by UV exposure. A complete healing was achieved in 4 weeks, with excellent integration and cosmetic appearance, as well as perfect visual acuity, while gentamicin sulfate (3%) eye drop instillations every two hours and total protection against UV exposure were prescribed. The choice of antibiotics is an important step in surgical management of pterygium, either as a single drug or a combination of fortified antibiotic therapy, in terms of efficacy in the endpoints of complete re-epithelialization.

## 5. Conclusions

Based on histological examination and complementary AFM and FTIR spectroscopy, we investigated the ultrastructural modification of the amniotic membrane upon gentamicin treatment and/or UV exposure, in the context of its suitability to be used as a graft in corneal reconstruction. The morphological features evidenced by H&E and immunohistochemical investigations were correlated with structural and ultrastructural modifications. The loss of cuboidal cells in the basal membrane was accompanied by the splitting of collagen fibers, which was in concordance with the structural alteration of collagen molecules, as evidenced by the FTIR quantitative analysis of the protein’s secondary structure. At the nano-scale, AFM details showed modifications of collagen fibrils in terms of their thickness and network compaction upon gentamicin and/or UV treatment. The collagenase digestion assay demonstrated that UV exposure significantly reduces the degradation rate of the AM, while gentamicin treatment promotes an accelerated enzymatic digestion of the AM upon UV exposure. Within the limitations of our study, the results might have importance in the context of the current debate over whether the AM should be used intact (fresh, preserved at −20 °C) or photo-crosslinked as a grafting material, taking into account the denaturation influenced by post-surgical antibiotic treatment.

## Data Availability

The data presented in this study are available on request from the corresponding author.

## Supplementary Materials

The following are available online at https://www.mdpi.com/1996-1944/14/4/863/s1: Figure S1: The AFM profiles of single collagen fibril in amniotic membrane after different treatments: (a) natural membrane (no treatment) (AMN); (b) membrane exposed to UV (AUV); (c) membrane exposed to gentamicin treatment 40 mg/mL (AG40); (d) membrane exposed to gentamicin treatment 80 mg/mL (AG80); (e) membrane exposed to gentamicin and UV treatment (AGUV).
