# Supplementary material for: Nano-Scale Modifications of Amniotic Membrane Induced by UV and Antibiotic Treatment: Histological, AFM and FTIR Spectroscopy Evidence
Source: Materials (Basel). 2021 Feb 11;14(4):863. doi: 10.3390/ma14040863 (PMC7917607; doi:10.3390/ma14040863)
Supplement: Supplementary file 1 [file materials-14-00863-s001.zip › materials-1053919-supplementary.pdf]

*materials*

*Supplementary Materials*

# Nano-Scale Modifications of Amniotic Membrane Induced by UV and Antibiotic Treatment: Histological, AFM and FTIR Spectroscopy Evidence

**Simona Cavalu [1], George Roiu [1,*], Ovidiu Pop [1,*], Denisa A. Petricas Heredea [1,*], Traian Octavian Costea [2] and Claudia Florida Costea [3]**

[1] Faculty of Medicine and Pharmacy, University of Oradea, 10 P-ta 1 Decembrie, 410073 Oradea, Romania; scavalu@uoradea.ro
[2] Advanced Materials Research Laboratory, University of Oradea, 1 University Street, 410087 Oradea, Romania; tcostea@uoradea.ro
[3] "G.T. Popa" University of Medicine and Pharmacy Iasi, 16 University Street, 700115 Iasi, Romania; claudia.costea@umfiasi.ro
* Correspondence: george.roiu@didactic.uoradea.ro (G.R.); popo@uoradea.ro (O.P.); heredea.amaliadenisa@student.uoradea.ro (D.A.P.H.)

**Citation:** Cavalu, S.; Roiu, G.; Pop, O.; Petricas Heredea, D.A.; Costea, T.O.; Costea, C.F. Nano-Scale Modifications of Amniotic Membrane Induced by UV and Antibiotic Treatment: Histological, AFM and FTIR Spectroscopy Evidence. *Materials* **2021**, *14*, 863. https://doi.org/10.3390/ma14040863

Academic Editor: Francesco Baino
Received: 11 December 2020
Accepted: 9 February 2021
Published: 11 February 2021

**Publisher's Note:** MDPI stays neutral with regard to jurisdictional claims in published maps and institutional affiliations.

**Copyright:** © 2021 by the authors. Licensee MDPI, Basel, Switzerland. This article is an open access article distributed under the terms and conditions of the Creative Commons Attribution (CC BY) license (http://creativecommons.org/licenses/by/4.0/).

The profiles of single collagen fibril exposed on the surface of amniotic membrane after different treatments are presented below.

**(a)**

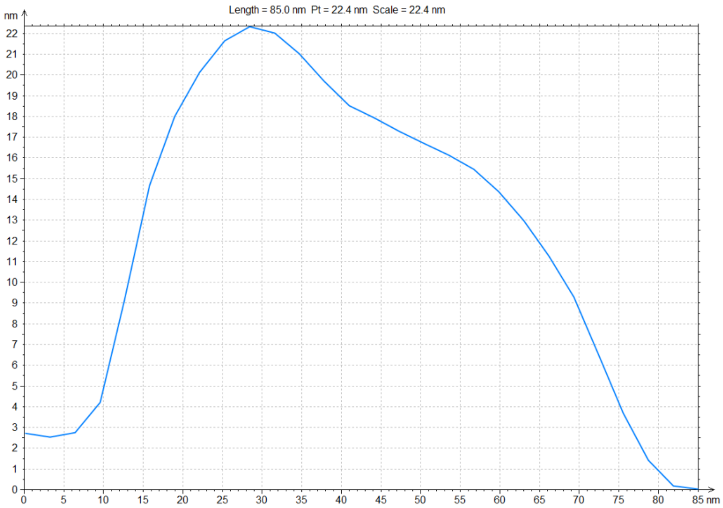

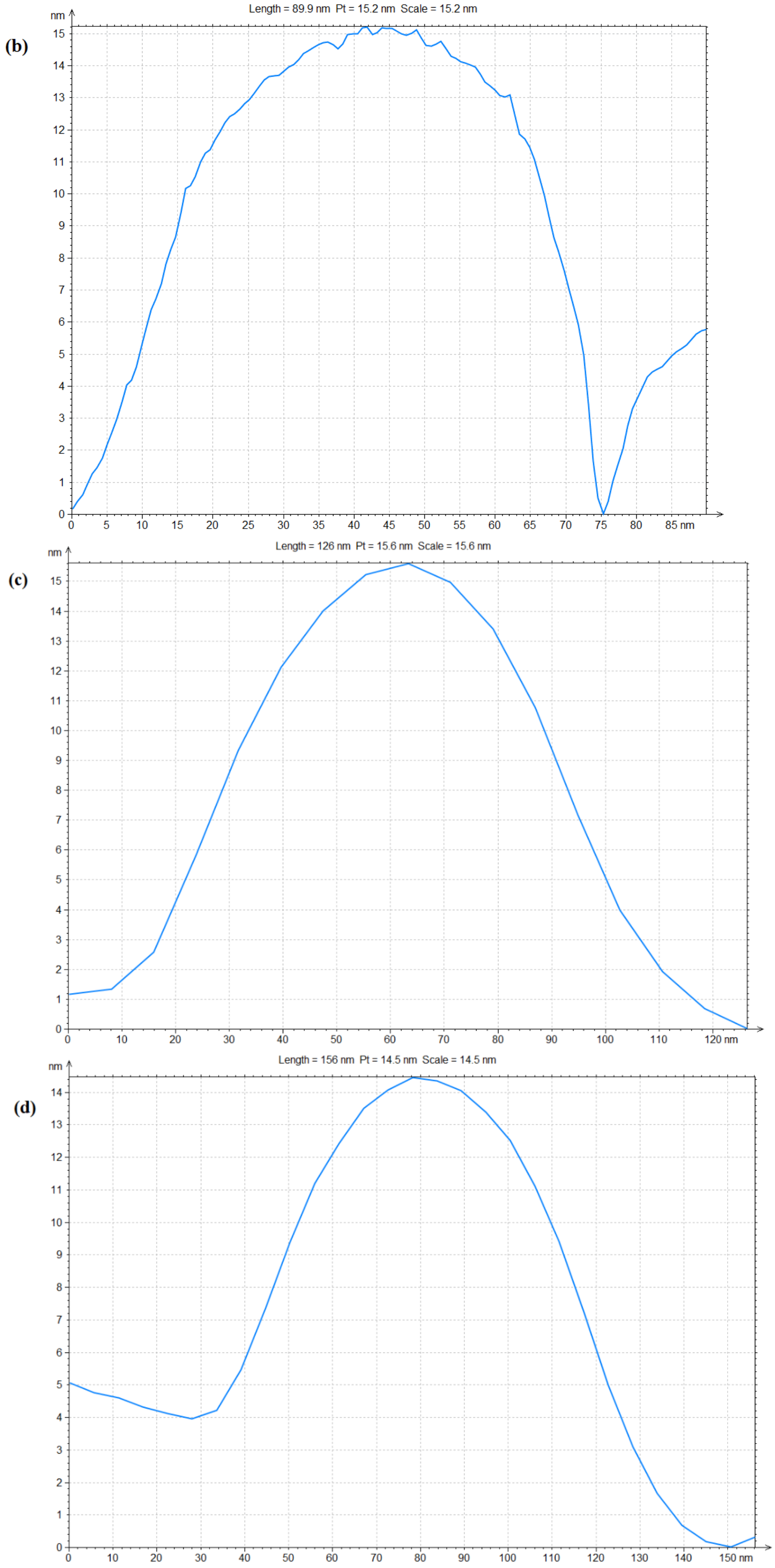
(b)
Length = 89.9 nm Pt = 15.2 nm Scale = 15.2 nm
nm
(c)
Length = 126 nm Pt = 15.6 nm Scale = 15.6 nm
nm
(d)
Length = 156 nm Pt = 14.5 nm Scale = 14.5 nm
nm

(e)

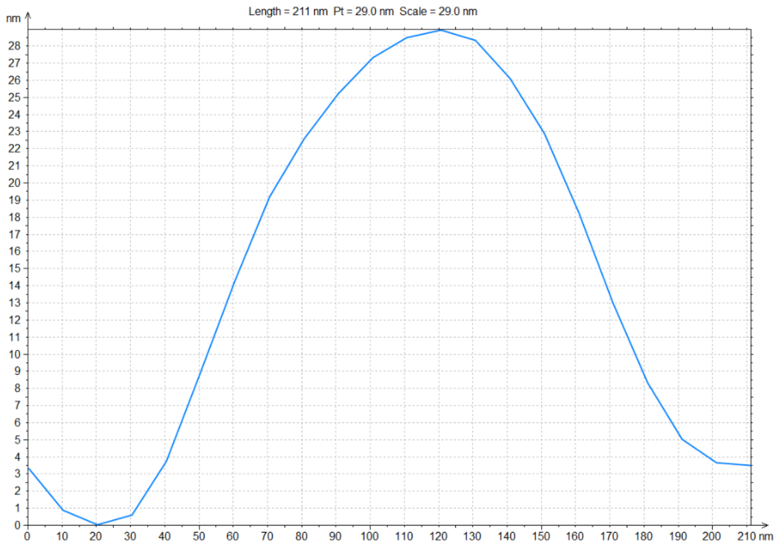


**Figure S1.** The AFM profiles of single collagen fibril in amniotic membrane after different treatments: (**a**) natural membrane (no treatment) (AMN); (**b**) membrane exposed to UV (AUV); (**c**) membrane exposed to gentamicin treatment 40 mg/ml (AG40); (**d**) membrane exposed to gentamicin treatment 80 mg/ml (AG80); (**e**) membrane exposed to gentamicin and UV treatment (AGUV).
